# Weekly group tummy time classes are feasible and acceptable to mothers with infants: a pilot cluster randomized controlled trial

**DOI:** 10.1186/s40814-020-00695-x

**Published:** 2020-10-14

**Authors:** Lyndel Hewitt, Samantha Stephens, Abbe Spencer, Rebecca M. Stanley, Anthony D. Okely

**Affiliations:** 1 Early Start, Faculty of Social Sciences, University of Wollongong, Wollongong, New South Wales 2522 Australia; 2 Illawarra Health and Medical Research Institute, Wollongong, New South Wales 2500 Australia; 3 Illawarra Shoalhaven Local Health District, Illawarra and Shoalhaven Regions, New South Wales Australia

**Keywords:** Tummy time, Infant, Objective, Accelerometer, Group, Physical activity, WhatsApp®

## Abstract

**Background:**

The World Health Organization recommends 30 min of tummy time daily for improved motor development and reduced likelihood of plagiocephaly. As only 30% of infants meet this recommendation, parents require strategies and support to increase this proportion.

**Methods:**

The aim of this study was to determine the feasibility, acceptability, and potential efficacy of a group intervention to promote tummy time. The design is a cluster randomized controlled trial with concealed allocation, assessor blinding, and intention-to-treat analysis. Five groups of healthy infants (*N* = 35, baseline mean (SD) age 5.9 (2.8) weeks) and their mothers attending local mother’s groups (Australia) were randomly allocated to the intervention or control group. The intervention group received group tummy time classes in addition to usual care. The control group received usual care with their child and family health nurse. Primary outcomes were intervention feasibility and acceptability. Secondary outcomes were tummy time duration (accelerometry), adherence to physical activity guidelines, head shape, and motor development. Measures were taken at baseline, post-intervention, and when infants were 6 months of age. Analyses were by linear mixed models and Cohen’s *d* statistic.

**Results:**

Recruitment, retention, and collection of objective data met feasibility targets. Acceptability was also met with intervention mothers reporting the information, goal planning, and handouts significantly more useful and relevant than control group mothers (*p* < 0.01). Moderate effect sizes were also found at post-intervention for tummy time duration, adherence to physical activity guidelines and infant ability in prone and supine favoring the intervention group (intervention infants had a mean of 30 min and 30% adherence to guidelines (95% CI 0 to 60.6 min) compared to the control infants who had a mean of 16.6 min and 13% adherence to the guidelines (95% CI 0 to 42.1 min, Cohen’s *d* = 0.5). Limitations were the small sample size, 4-week intervention, limited accelerometer use, and a homogenous sample of participants.

**Conclusion:**

Group tummy time classes delivered in a mother’s group setting were shown to be feasible and acceptable. A larger randomized controlled trial is warranted.

**Trial registration:**

ANZCTR, ACTRN12617001298303p. Registered 11 September 2017

## Key messages regarding feasibility

- *What uncertainties existed regarding the feasibility?* The feasibility of a practical group exercise (tummy time) class conducted in mother’s groups is currently unknown. In addition, the feasibility of objectively measuring tummy time using an accelerometer in a real-world setting is also unknown.
- *What are the key feasibility findings?* Thirty-five mothers (83%) were recruited to this study and there were two drop outs (94% retention baseline to post-intervention). The use of GENEActiv to measure tummy time was acceptable to 80% of mothers overall (post-intervention, follow-up); however, 45% (post-intervention) reported the accelerometer required input throughout the day to keep it positioned correctly.
- *What are the implications of the feasibility findings for the design of the main study?* The intervention was well received by the mothers in the mother’s groups. Mothers require support and reminders to use the accelerometer on their baby for more than 2 days. Future trials should recruit from more than one local health district to obtain a more diverse sample of participants.

## Background

The World Health Organization (WHO) recommends infants are physically active several times a day in a variety of ways for the greatest health benefits. This includes at least 30 min in a prone position (tummy time) spread throughout the day while they are awake and supervised [1]. Benefits of tummy time include improved motor development [2] and a reduced likelihood of deformational plagiocephaly [3]. These recommendations also form part of the 24-h Movement Guidelines for Australia, Canada, South Africa, and the UK [4–7].

Despite the implementation of interventions aimed at improving infant nutrition, physical activity, and sedentary behavior [8, 9], only 30% of parents adhere to these physical activity (tummy time) recommendations [10]. Interventions aimed to increase awareness, translation and implementation of infant physical activity guidelines are required to determine which strategies and types of support given to parents/carers are feasible, acceptable, and efficacious. Assisting parents and carers to meet the recommended targets will ensure the achievement of optimal health and development outcomes for their children.

The purpose of this study was to develop, implement, and test an intervention to determine the feasibility, acceptability, and potential efficacy of the intervention to promote tummy time when delivered in a mother’s group setting.

The research questions for this study were:

1. What is the feasibility of a group intervention to promote tummy time when delivered in a mother’s group setting?
2. What is the acceptability of a group intervention delivered to mothers to promote tummy time when delivered in a mother’s group setting?
3. What is the potential efficacy (tummy time duration, adherence to infant physical activity guidelines, head shape, motor development) of a group intervention to promote tummy time when delivered in a mother’s group setting?

## Methods

### Overview

This study investigated the feasibility and acceptability of group tummy time classes to mothers and their infants who were attending their local area health service mother’s groups. The primary outcome was feasibility and acceptability of the intervention. The secondary outcomes were tummy time duration, adherence to the tummy time guidelines for infants, the infant’s head shape, and motor development. This study complied with the CONsolidated Standards Of Reporting Trials (CONSORT) statement. The University of Wollongong and Illawarra Shoalhaven Local Health District Health and Medical Human Research Ethics Committee approved the study (2017/368). This trial was prospectively registered in September 2017 with the Australian New Zealand Clinical Trials Registry (ANZCTR). Written informed consent was obtained by the infant’s mother on behalf of themselves and the infant prior to the commencement of the study. This study design will be recommended for further investigation if found to be adequately feasible and acceptable (positive outcomes for the intervention usefulness and acceptability) to mothers attending their local area health district mother’s groups. Based on previous studies [11, 12], feasibility will be assessed against the following: (1) more than 30 participants to be screened and recruited, (2) 80% of participants would be retained, and (3) 70% of objectively measured physical activity (tummy time) would be successfully collected at baseline and follow-up. Acceptability will be assessed against these parameters: (1) 100% of the planned sessions would be implemented, (2) there would be a minimum 80% attendance and 80% use of WhatsApp® overall, and (3) more than 80% of the intervention participants would report the intervention to be acceptable and useful based on parent survey.

### Participants and recruitment

Participants were recruited in groups from an Early Childhood Health Service in New South Wales (NSW), Australia (January 2018 to September 2018). Mothers with infants in the post-natal period (birth to 12 weeks) are usually placed into groups by the early childhood health nurse (ECHN) from this health service. Mothers (and their infants) attending these groups were invited to participate in the study by the principal researcher. The inclusion criteria were a mother and her healthy infant who would attend their local mother’s group weekly for 4 weeks. Originally, the study was to include first time mothers only. However, this was too exclusive in a small group context and consequently all mothers in the group were invited to participate.

### Sample size, group randomization, and blinding

No sample size power calculations were taken as the main outcome of this study was feasibility [13]. However, the sample size was determined to provide information that would be sufficient to demonstrate feasibility. The final sample size is commensurate with many pilot studies in this area [11]. Whole groups of participants were randomized to intervention or usual care (control) after baseline data collection. Randomization of the group to receive the intervention or usual care (control group) was determined by a computer-generated random numbers program, conducted by a researcher who was not part of the research team and was blinded to group allocation and all participant information. The infant outcome measurements (baseline, post-intervention, follow-up) were taken by a member of the research team who was blinded to the group allocation. To avoid observer effect (Hawthorne effect), mothers were not told the accelerometer was specifically measuring the amount of tummy time. They were only told the accelerometer was measuring the infant’s physical activity.

### Intervention

Bandura’s Social Cognitive Theory was used to develop an intervention that considered how personal, behavioral, and environmental factors influence the amount of tummy time given to an infant [14, 15]. The intervention was integrated into the start of the mother’s group’s usual 2-h session with their early childhood nurse. Personal factors (e.g., importance of tummy time) were addressed by an educational component in the intervention that aimed to improve the value and benefits of tummy time. Behavioral (e.g., setting aside time) and environmental factors (e.g., organizing a space and having equipment ready) were also addressed by demonstrating and discussing techniques and equipment for achieving tummy time throughout the day. Small, achievable goals were set each week to assist mothers with the frequency and duration of tummy time. Discussions were had the week following to allow mothers to converse with their peers regarding tips and strategies they found helpful or to allay any concerns. Most importantly, tummy time was practiced together at the mother’s groups. This gave practical experience with immediate feedback and encouragement, enabling mothers to have the confidence to attempt tummy time at home. The intervention was conducted by a physiotherapist (principal researcher) and was designed to improve parent self-efficacy (persuasion and experience) and mastery (attention, retention, goal setting, perceived barriers, practicing together and motivation) (Additional file 1).

A WhatsApp® social media group was also included in the intervention group to provide an outlet for mothers to encourage each other regarding strategies they found helpful during the week regarding their infant’s tummy time. Messaging via WhatsApp® was limited to the 4-week intervention time only and was not continued after this time. Standardized messages were sent from the principal researcher via WhatsApp® three times per week encouraging the mothers to practice tummy time with their infant. For example, “Hi Mums! Hope you are having a lovely day. If you can, try to meet our goal today of 3 sessions of tummy time today. Don’t forget to use a timer. Have fun and let me know how you go!  ”. Standardized replies were also sent after a mother posted a message about their baby’s tummy time. For example, “Yay! Well done [baby name]!  ”.

The use of text messaging and social media platforms to enhance healthy behavior is also an emerging field of research. Positive benefits have been shown for a parent text messaging intervention aimed to reduce sedentary behavior in young children (ages 2 to 4 years) [13]. The use of a parent delivered social media messaging system to influence infant behavior is yet to be investigated.

### Usual care

Groups randomized to the control group received usual care, which was to attend their local area health service mother’s group sessions (once per week for four weeks). During these sessions, topics such as breastfeeding, settling, tummy time, and development for example were discussed on an ad hoc basis. The sessions were conducted by the Early Childhood Nurse Practitioner from this health service. A mother in each control group set up her own social media group for the mothers in her respective group to enable social interaction and organization of events as required.

### Outcome measures

Data were collected at baseline, post-intervention and again when the infants were approximately 6 months old (follow-up).

### Primary outcomes

#### Feasibility and acceptability

The number of mothers recruited to the study, retention during the study, and the number that consented to using the GENEActiv to measure their infant’s physical activity was used to determine intervention feasibility. Recruitment was measured by the percentage of mothers who consented to be a part of the study. Retention was determined by the percentage of mothers who completed post-intervention measurements. Feasibility of the GENEActiv to measure physical activity was measured by calculating the amount of useable data that were collected. Intervention acceptability was measured by the number of sessions that were implemented, the attendance of the participants at the mother’s groups, and the number of mothers who chose to participate with the WhatsApp® messaging. In addition, a post-intervention parent questionnaire (usefulness and relevance) was given to all intervention and control groups [13] (Additional file 2).

### Secondary outcomes

#### Tummy time

Tummy time duration (minutes) was measured using the GENEActiv accelerometer which has been validated in a previous study [16]. All GENEActiv data were included in the baseline and post-intervention measurement time points as all mothers reported their infants were supine sleeping (sleeping on their back). When the infants were 6 months old, it was reported that 15% were sleeping prone (sleeping on their tummy, 10% from the intervention group, 18% from the control group). To account for this, nighttime and naptime sleep data were removed for all infants according to the parent questionnaire regarding their infant’s routine. Parents were asked to place the GENEActiv on their infants for 3, 24-h periods over 7 days to measure the amount of tummy time they received (baseline, post-intervention, follow-up). The GENEActiv is worn on the right hip secured by an elastic strap around the waist. The device was initialized at 30 Hz and data was collected in 1 s epochs as per the validation study [16]. Wear and non-wear time was classified by using temperature and z-axis cut points [17].

Previous tummy time studies have used subjective parent reports to provide information regarding the amount of time an infant engages in tummy time. These studies do not report the validity and reliability of the questionnaires used [18]. With the absence of an objective tool for comparison, the accuracy of these reports is unknown. The use of accelerometry has been demonstrated to be effective in determining child engagement in physical activity in older age groups [19]. This research is now extending to infant movement with the GENEActiv accelerometer (ActivInsights Ltd, UK) recently validated to determine the time spent in non-prone or prone positions by an infant as 98% and 95%, respectively [16].

#### Head shape and motor development

A physiotherapist (who was blinded to group allocation) with more than 20 years’ experience in pediatric care measured the infant outcomes (head shape, motor development). Head shape was measured using cranial calipers to calculate the cranial diagonal difference (CDD) to determine the presence of plagiocephaly and cephalic index (CI) to determine the presence of brachycephaly [20]. Motor development was assessed using the Alberta Infant Motor Scale (AIMS) [21]. The AIMS is designed to assess gross motor ability in infants from birth through to walking [22]. The scale has four categories: prone, supine, sitting and standing. Categories are divided into abilities relating to the final achievement of each skill. Each ability the infant achieves is scored as “observed” or “not observed” (1 point per ability). The reliability and validity of this scale is reported in previous studies [21].

#### Statistical analysis

Analyses were conducted using SPSS version 25 (IBM Corp, Armonk, NY, USA). Descriptive statistics were used to describe the demographic information of those participating in the study. Feasibility and acceptability were assessed using percentages and comparing between groups using Pearson Chi Square. Groups were compared by tummy time duration, head shape, and motor development using linear mixed models (adjusted for clustering by mother). As there was not adequate power to detect statistically significant differences, Cohen’s *d* was calculated to determine effect sizes. Cohen’s *d* values of approximately 0.20, 0.50, and ≥ 0.80 were defined as a small, moderate and large effect sizes, respectively [23]. Intention-to-treat principles were followed with all participants analyzed in the group in which they were randomized and irrespective of whether they finished the intervention. See Table 1 for further information.

**Table 1:** Outcome measure analysis methods

Outcome measure	Analysis method
Primary outcomes—feasibility and acceptability	
Recruitment and retention	Descriptive statistics (*n*, %)
Usefulness and relevance	Descriptive statistics (*n*, %), Pearson chi square
Engagement in social media	Descriptive statistics (*n*, %), Pearson chi square
Wearing the GENEActiv	Descriptive statistics (*n*, %)
Secondary outcomes—potential efficacy	
Amount of tummy time and adherence to the 24-h movement guidelines	Descriptive statistics (*n*, %), linear mixed models (group, time, group × time, sex), Cohen’s *d*
Motor development and head shape	Descriptive statistics (*n*, %), linear mixed models (group, time, group × time, sex), Cohen’s *d*
Personal, behavioral, and environmental factors	Descriptive statistics (*n*, %), linear mixed models (group, time, group × time, sex), Cohen’s *d*

*n* number, *%* percentage

## Results

### Demographic information

Demographic information is presented in Table 2. Two mothers and their infants from the intervention group did not attend any intervention sessions despite consenting to participate at baseline. One mother was not contactable after baseline measures and the other declined to participate due to wanting to prioritize receiving assistance for breastfeeding difficulties. There were no unintended effects or harms caused to any of the participants. This was assessed by the physiotherapist during their outcome assessments.

**Table 2:** Demographic information

		Intervention group, *n* = 16	Control group, *n* = 19
Male	*n* (%)	10 (62.5%)	8 (42.1%)
Maternity country of birth (Australia)	*n* (%)	13 (81.3%)	17 (89.5%)
Paternity country of birth (Australia)	*n* (%)	15 (93.8%)	18 (94.7%)
Household income (> $80000)	*n* (%)	13 (81.3%)	16 (84.2%)
Age of mother (years)	*M* (SD)	33 (3.5)	32 (4.5)
Highest level of education completed by mother (university or higher)	*n* (%)	14 (87.5%)	16 (84.2%)
Birth weight (kg)	*M* (SD)	3.3 (0.4)	3.4 (0.4)
Birth length (cm)	*M* (SD)	50.4 (1.7)	51 (2.4)
Medical conditions that affect functional ability	*n*	0	0
Gestational age (weeks)	*M* (SD)	38.6 (0.9)	39.1 (1.2)
Age at which tummy time was started (weeks)	*M* (SD)	2.3 (1.3)	2.1 (1.7)
Age (baseline) (weeks)	*M* (SD)	5.6 (2.3)	6.2 (3.3)
Age (post-intervention) (weeks)	*M* (SD)	11.1 (2.9)	10.2 (3.2)
Age (follow-up) (months)	*M* (SD)	6 (0.6)	5.6 (1.0)

*M* mean, *SD* standard deviation, *n* number, *%* percentage

### Primary outcomes—feasibility and acceptability

#### Recruitment and retention

**Fig. 1:**
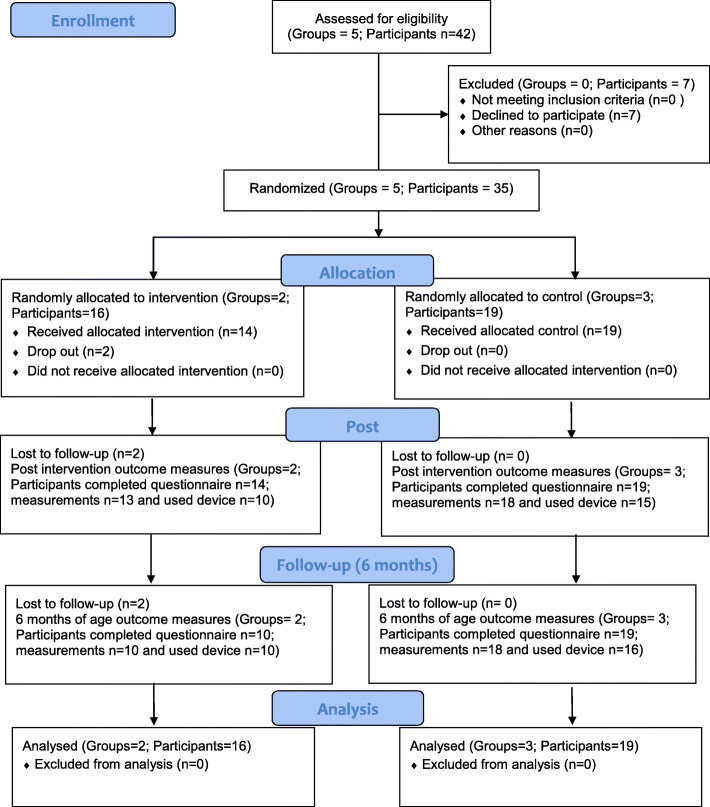
CONSORT flow diagram

Forty-two mothers were asked to partake in the study from five different mother’s groups. Thirty-five mothers (83%) consented to participate in the study and were randomized (by group) to either the intervention (*n* = 16) or control group (*n* = 19). There were two intervention groups and three control groups with an average of seven mother/infant pairs per group (range 6 to 9 mother/infant pairs per group). Attendance was on average 69% over the four sessions for the intervention group (includes drop outs) and 80% for the control group. Figure 1 demonstrates the flow of participants through the study [24] (see Additional file 3 for CONSORT checklist [24]) and Table 3 has the attendance record of participants in their respective sessions. There were no significant differences between the drop outs (*p* > 0.05 for all baseline variables for mothers and infants) and the remaining participants in the study.

**Table 3:** Attendance in mother’s group sessions, *n* (%)

	Intervention group (*n* = 16^a^)	Control group (*n* = 19)	Total (*N* = 35)
Session 1	12 (75)	19 (100)	31 (88.6)
Session 2	10 (62.5)	16 (84.2)	26 (74.3)
Session 3	10 (62.5)	14 (73.7)	24 (68.6)
Session 4	12 (75)	12 (63.2)	24 (68.6)

^a^Includes two drop outs from intervention group after baseline measures

#### Usefulness and relevance

**Fig. 2:**
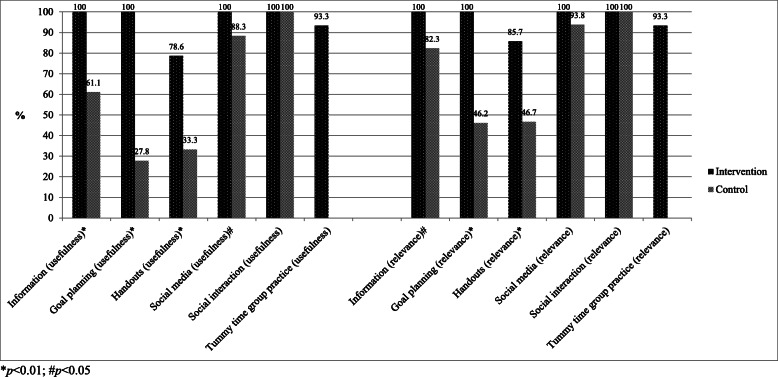
Usefulness and relevance of intervention and control group programs (extremely or very). *p < 0.01, ^#^*p* < 0.05

All mothers in the intervention group reported that the information and goal planning was extremely or very useful compared with 61% (information) and 28% (goal planning) in the control group (*p* < 0.01). Similarly, 100% of mothers in the intervention group reported that the information and goal planning was extremely or very relevant compared with 82% (information, *p* < 0.05) and 46% (goal planning, *p* < 0.01) in the control group. Ninety-three percent of the intervention group mothers thought the tummy time group practice was extremely or very useful (Fig. 2).

#### Engagement in WhatsApp®

For the intervention group, standardized messages were sent three times per week (12 messages per intervention group) from the principal researcher encouraging the mothers to practice tummy time with their infant. In addition, there were 65 standardized replies to the 65 messages sent by mothers about their own baby’s tummy time. Approximately 88% of intervention mothers participated in WhatsApp® and sent messages to their group. There were 128 messages (average of 9 messages per mother) sent that were relevant to tummy time (104 not relevant). Of the messages that were relevant to tummy time, 51% of those messages were about their own baby and 49% were messages of encouragement to another mother. For the control group, 84% of mothers reported they participated in a social media group set up by one of the mothers in their group. All three control groups chose Facebook as their social media platform to communicate with each other for the purpose of social interaction. Both the intervention and control groups reported the social media was relevant to them equally (*p* > 0.05); however, the intervention mothers reported the social media was significantly more useful than the control group mothers (*p* < 0.05) (Fig. 2).

#### Wearing the GENEActiv

Thirty-one mothers (89%), 25 mothers (71%), and 26 mothers (77%) consented to use the accelerometer to measure their infant’s physical activity at baseline, post-intervention, and at follow-up, respectively. Use of the GENEActiv to measure tummy time was feasible, with most infants wearing the device for approximately two days, with a range from 0 to 4 days (calculated from GENEActiv data). One (intervention group), two (one infant each from the intervention and control groups), and four infants (two each from the intervention and control groups) had less than 24-h wear time at baseline, post-intervention, and follow-up, respectively. These infants were not excluded from the analysis due to the already small sample size. Additional analysis (linear mixed models) revealed this made little difference to the final outcome. A practicality questionnaire was also administered at each measurement time point. The outcomes of the practicality questionnaire are reported in Table 4.

**Table 4:** Practicality questionnaire—number of parents and percentage of agreement

The device	Post-intervention (*n* = 25)	Follow-up (*n* = 26)
Did not interfere with the positions my baby was placed in	22 (88%)	20 (77%)
Did not interfere with my baby’s ability to move around freely	21 (84%)	23 (88%)
Was not uncomfortable for my baby to wear (including attaching and removing devices)	21 (84%)	21 (81%)
Did not require a lot of input to ensure the device was kept on correctly	14 (56%)	16 (61%)
Was able to be attached by myself	23 (92%)	26 (100%)
Could be tolerated by my baby to wear (during the daytime) for at least 3 days	22 (88%)	23 (88%)

Practicality questionnaire results presented as number (*n*) and percentages (%)

### Secondary outcomes—potential efficacy

#### Amount of tummy time and adherence to 24-h movement guidelines

**Fig. 3:**
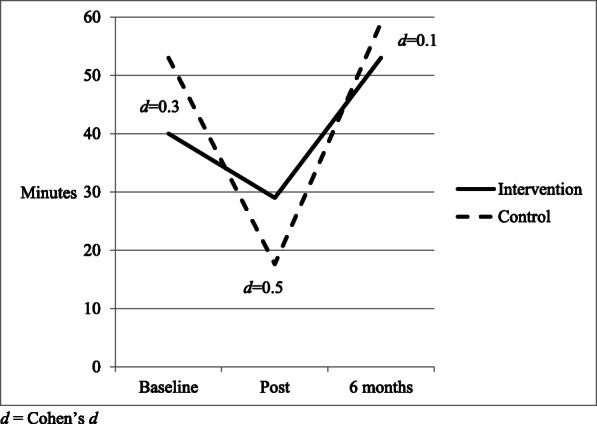
Mean duration of tummy time per day (minutes). *d*, Cohen’s *d*

**Fig. 4:**
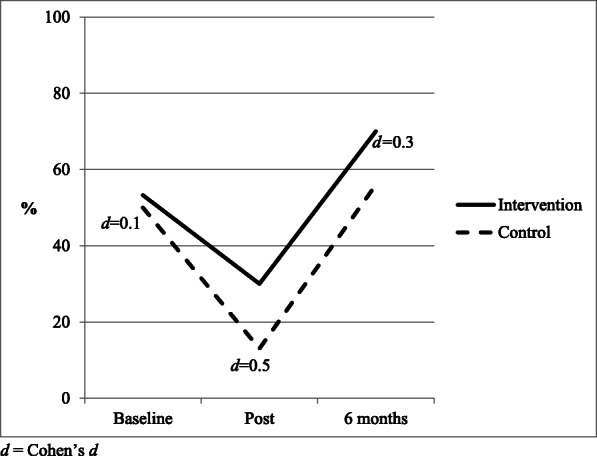
Percentage of infants meeting physical activity guidelines. *d*, Cohen’s *d*

There were no overall differences between the groups regarding the amount of tummy time the baby received (control group 95% CI, 26.9 to 58.5 min; intervention group 95% CI, 21.9 to 59 min, *p* = 0.623) from the linear mixed models analysis. However, there was a moderate effect size favoring the intervention group for both tummy time duration and percentage adhering to guidelines at the post-intervention measurement (Cohen’s *d* = 0.5). At post-intervention, control babies had a mean of 16.6 min and 13% adherence to the guidelines (95% CI 0 to 42.1 min), whereas the intervention babies had a mean of 30 min and 30% adherence to guidelines (95% CI 0 to 60.6 min, Cohen’s *d* = 0.5). At 6 months of age, a small effect size was found with 70% of the intervention group meeting the guidelines compared with 56% of the control group (*d* = 0.3) (Figs. 3 and 4).

#### Motor development and head shape

There were no differences between groups regarding motor development (control group 95% CI, 12.3 to 14.7 AIMS total points; intervention group 95% CI, 11.5 to 14.4 AIMS total points, *p* = 0.691). There was a moderate effect size at post-intervention for the infant’s ability in prone (*d* = 0.5) and sitting (*d* = 0.7) favoring the intervention group (Table 5). There was a significant effect on the cranial index (*p* = 0.012) with post hoc analysis showing that the cranial index increases for the intervention group only from baseline (81.8%) to post-intervention (85.7%) (*p* = 0.001). There was also one extreme outlier in the intervention group in regards to cranial index and as such was removed from the head shape analysis. There were no differences between the groups for the plagiocephaly head shape measure (control group 95% CI, 2.4 to 3.8 mm; intervention group 95% CI, 1.5 to 3.1 mm, *p* = 0.314) from the linear mixed models.

**Table 5:** Tummy time per day (min), percentage of infants meeting 24-h movement guidelines (tummy time), motor development scores (AIMS), and head shape (mm)

	Intervention group (*N* = 16)	Control group (*N* = 19)	Total (*N* = 35)	Cohen’s *d*
Tummy time per day in min, mean (95% CI)				
Baseline	40.0 (14.9–64.2) (*n* = 15)	52.4 (28.6–76.1) (*n* = 16)	46.0 (28.8–63.1) (*n* = 31)	0.3
Post-intervention	30.3 (0.0–60.6) (*n* = 10)	16.6 (0.0–42.1) (*n* = 15)	23.5 (3.6–43.3) (*n* = 25)	0.5
6 months old	51.5 (21.2–81.9) (*n* = 10)	59.1 (35.4–82.9) (*n* = 16)	55.3 (36.1–74.6) (*n* = 26)	0.1
Met recommendations (%)				
Baseline	53.8% (*n* = 15)	50.0% (*n* = 16)	51.9% (*n* = 31)	0.01
Post-intervention	30% (*n* = 10)	13.3% (*n* = 15)	21.7% (*n* = 25)	0.5
6 months old	70% (*n* = 10)	56.3% (*n* = 16)	63.2% (*n* = 26)	0.3
AIMS scores, mean (95% CI)				
Baseline	*n* = 16	*n* = 18	*n* = 34	
Prone	2.3 (1.3–3.3)	2.3 (1.4–3.3)	2.3 (1.7–3.0)	0.0
Supine	2.4 (1.9–3.0)	2.6 (2.1–3.1)	2.5 (2.1–2.9)	0.3
Sitting	1.0 (0.4–1.6)	1.2 (0.6–1.8)	1.1 (0.7–1.5)	0.3
Standing	1.0 (0.7–1.3)	1.2 (1.0–1.5)	1.1 (0.9–1.3)	0.5
Total	6.7 (4.7–8.7)	7.3 (5.4–9.2)	7.0 (5.6–8.4)	0.3
Post-intervention	*n* = 13	*n* = 18	*n* = 31	
Prone	3.5 (2.5–4.6)	3.2 (2.2–4.1)	3.4 (2.6–4.1)	0.5
Supine	3.4 (2.8–4.0)	3.6 (3.2–4.1)	3.5 (3.1–3.9)	0.5
Sitting	1.8 (1.1–2.5)	1.4 (0.8–2.0)	1.6 (1.2–2.1)	0.7
Standing	1.5 (1.2–1.8)	1.6 (1.3–1.8)	1.5 (1.4–1.7)	0.0
Total	10.3 (8.0–12.5)	9.8 (7.9–11.7)	10.0 (8.6–11.5)	0.2
6 months old	*n* = 10	*n* = 17	*n* = 27	
Prone	7.8 (6.6–9.0)	8.7 (7.8–9.7)	8.3 (7.5–9.0)	0.3
Supine	6.9 (6.2–7.6)	6.9 (6.4–7.4)	6.9 (6.5–7.3)	0.1
Sitting	5.1 (4.3–5.8)	5.3 (4.7–5.9)	5.2 (4.7–5.7)	0.1
Standing	2.2 (1.9–2.5)	2.5 (2.2–2.7)	2.3 (2.1–2.5)	0.5
Total	21.9 (19.4–24.5)	23.4 (21.5–25.4)	22.7 (21.1–24.3)	0.2
Head shape, mean (95% CI)				
Baseline	*n* = 15	*n* = 18	*n* = 34	
CDD (mm)	3.1 (2.0–4.1)	3.6 (2.6–4.5)	3.3 (2.6–4.0)	0.1
CI (%)	81.8 (78.8–84.9)	82.6 (79.8–85.5)	82.2 (80.2–84.3)	0.1
Post-intervention	*n* = 12	*n* = 18	*n* = 31	
CDD (mm)	1.7 (0.5–2.9)	3.4 (2.4–4.4)	2.5 (1.8–3.3)	0.7
CI (%)	85.7* (82.5–88.8)	82.5 (79.6–85.3)	84.1 (82.0–86.2)	0.6
6 months old	*n* = 9	*n* = 17	*n* = 27	
CDD (mm)	2.0 (0.7–3.4)	2.4 (1.4–3.4)	2.2 (1.4–3.0)	0.0
CI (%)	84.8 (81.6–88.1)	82.8 (80.0–85.7)	83.8 (81.7–86.0)	0.2

*AIMS* Alberta Infant Motor Scale, *CDD* cranial diagonal difference, *CI* Cranial Index; *95% CI* 95% confidence interval

*p = 0.012 intervention group baseline to post-intervention measures only

#### Personal, behavioral, and environmental factors

There were no significant difference between groups at any measurement time point regarding the mothers reported knowledge about the benefits of tummy time, being afraid when their infant is on their tummy, tummy time being important to them, equipment used to assist their baby’s tummy time, setting aside time for their baby to spend on their tummy, use of various equipment to assist their tummy time sessions, and where tummy time usually takes place (all *p* > 0.05). Despite this, only the mothers in the intervention group (50%) reported they used a timer to assist in measuring the amount their baby was receiving.

## Discussion

This study investigated the feasibility, acceptability, and potential efficacy of group tummy time classes to mothers attending their local area health service mother’s groups. The content and group tummy time practice was feasible and acceptable to mothers. In addition, the use of a social media platform such as WhatsApp® was also useful for the mothers in this study to improve their compliance to tummy time recommendations. Using the accelerometer on infants as a measurement tool was somewhat feasible with an average wear time of approximately two days at each measurement time point. The effect of the program on the potential efficacy were promising at post-intervention with moderate effect sizes for tummy time duration, percentage meeting the guidelines, and ability in prone and sitting. The significant effect found for the cranial index measure from baseline to post-intervention was also an interesting finding; however, a larger RCT adequately powered to determine statistically significant differences would be required to make further recommendations regarding this information.

Designing a program taking into account many of the personal, behavioral and environmental factors affecting behavior change are shown to be more effective [25–29]. It is presumed that this is why the intervention was feasible and acceptable for the mothers in this study. The intervention was personal, included realistic goal setting and a supportive environment of mothers in the same stage of life. The effect sizes at 6 months of age were smaller than expected; this may be due to contact between mothers and researchers ceasing after the post-intervention measurements. The addition of a booster education session or the continuation of WhatsApp® messaging after the 4-week intervention may have assisted intervention mothers continue with their tummy time practices post-intervention [13].

Previous studies have also demonstrated an association between awake prone time and a better attainment of sitting skills [30, 31]. It is hypothesized that prone positioning improves trunk extension and upper limb weight bearing which are abilities that assist with learning to sit [31]. As experienced in this study, developing a relationship of trust with the parent is an important element in changing behavior [32]. Communicating directly and openly with families, health care professionals have opportunities to address concerns that may serve as barriers to providing prone playtime. As early childhood nurses (ECHN) care for parents and infants in this post-natal stage, the addition of 24-h Movement Guideline information to existing mother’s groups conducted by ECHN teams may be an ideal time to begin this education.

Tummy time remains a challenging task for parents. This study is important as it highlights that programs in the post-natal stage are required to assist parents meet the recommended targets. Parents in this study valued the practical, supportive assistance to encourage prone play. The use of group tummy time practice and starting a WhatsApp® group are two strategies that could be considered by ECHN teams to further support parents achieve optimal outcomes for their infants.

Translating current evidence to parents and health care providers about infant physical activity in a real-world setting and improving the ability to measure infant physical activity using objective measurement techniques are essential to target. Results from this study can be used to design a larger randomized controlled trial, refine other existing programs, and prioritize strategies that will optimize infant care through which positive health strategies can be started from birth.

Strengths of this study include the implementation of an intervention that was added to an existing health service aimed to assist mothers and their infants reach optimum health outcomes. Studies have demonstrated that starting early, role modeling, practicing skills, and building social networks are successful to enhance infant physical activity [33]. Additional strengths of the study were the use of objective measurement techniques, and the development of the intervention using Social Cognitive Theory. Limitations for this study include the small sample size, one area health service, a 4-week intervention, and limited number of days using the accelerometer to measure tummy time. Additionally, many mothers (approximately 45%) reported the accelerometer required input throughout the day to keep it positioned correctly. The use of a pouch to contain the accelerometer in clothing or inserting belt loops to a “wondersuit” or onesie may have decreased the burden on mothers to keep the accelerometer correctly positioned on the right hip. The groups were homogenous with mothers being mostly highly educated (> 85% with a university degree or higher), Australian, and from middle-income families. Results therefore from this study may not be transferrable to parents from lower socioeconomic backgrounds or from different cultural groups as the information was presented in English and required the use of a smart phone and dependent upon mothers attending their local mother’s groups for support. Control groups were also not monitored for the amount of tummy time education or practice that was discussed with the mothers in these groups even though it was usual practice for mother’s groups in this health district to include this information (informal ad hoc education). It is recommended that any future trials using this model of care attempt to recruit participants from more than one local health district to obtain a more diverse sample of participants. In addition, future trials should ensure GENEActiv placement is secured by belt loops and not dependent upon mothers to keep it positioned correctly, and obtain a larger sample size. As parenting groups are usually delivered by the ECHN, training nurses to implement this program would also assist with scale-up into the NSW Health system. Lastly, information regarding parent’s expectations of the mother’s groups would be important to consider in a larger randomized controlled trial to determine their pre-conceived ideas regarding the level of support or education that will be provided. Future trials may consider further piloting to determine the efficacy of the intervention being delivered by ECHN and the feasibility and acceptability of recruiting from multiple health districts.

## Conclusion

The addition of a group tummy time program was found to be feasible and acceptable to mothers attending their local area health service mother’s groups. WhatsApp® was also useful for the mothers in this study to improve their compliance to tummy time recommendations. Potential efficacy on motor development was positive for immediate effects (prone and sitting); however, overall effects remain inconclusive. A larger randomized controlled trial is warranted.

### Practical implications

- A group tummy time program with practical and social media components are feasible and acceptable to mothers attending their local area health service mothers groups.
- Providing support to parents in implementing the 24-h Movement Guidelines is needed in the infant stage of life.
- Objective measurement of tummy time by GENEActiv is feasible in the real-world setting.

## Supplementary information

**Additional file 1:** Bandura’s social cognitive theory – Intervention mapping

**Additional file 2:** Usefulness and relevance questionnaires

**Additional file 3:** CONSORT checklist

## Abbreviations

WHO: World Health Organization
CONSORT: CONsolidated Standards Of Reporting Trials
ANZCTR: Australian New Zealand Clinical Trials Registry
NSW: New South Wales
ECHN: Early childhood health nurse
CDD: Cranial diagonal difference
CI: Cranial index
AIMS: Alberta Infant Motor Scale
SPSS: Statistical Package for Social Sciences
CI: Confidence interval
