# Supplementary figures and images for: Weekly group tummy time classes are feasible and acceptable to mothers with infants: a pilot cluster randomized controlled trial
Source: Pilot Feasibility Stud. 2020 Oct 14;6:155. doi: 10.1186/s40814-020-00695-x (PMC7556919; doi:10.1186/s40814-020-00695-x)

### Additional file 1: Bandura's social cognitive theory – Intervention mapping

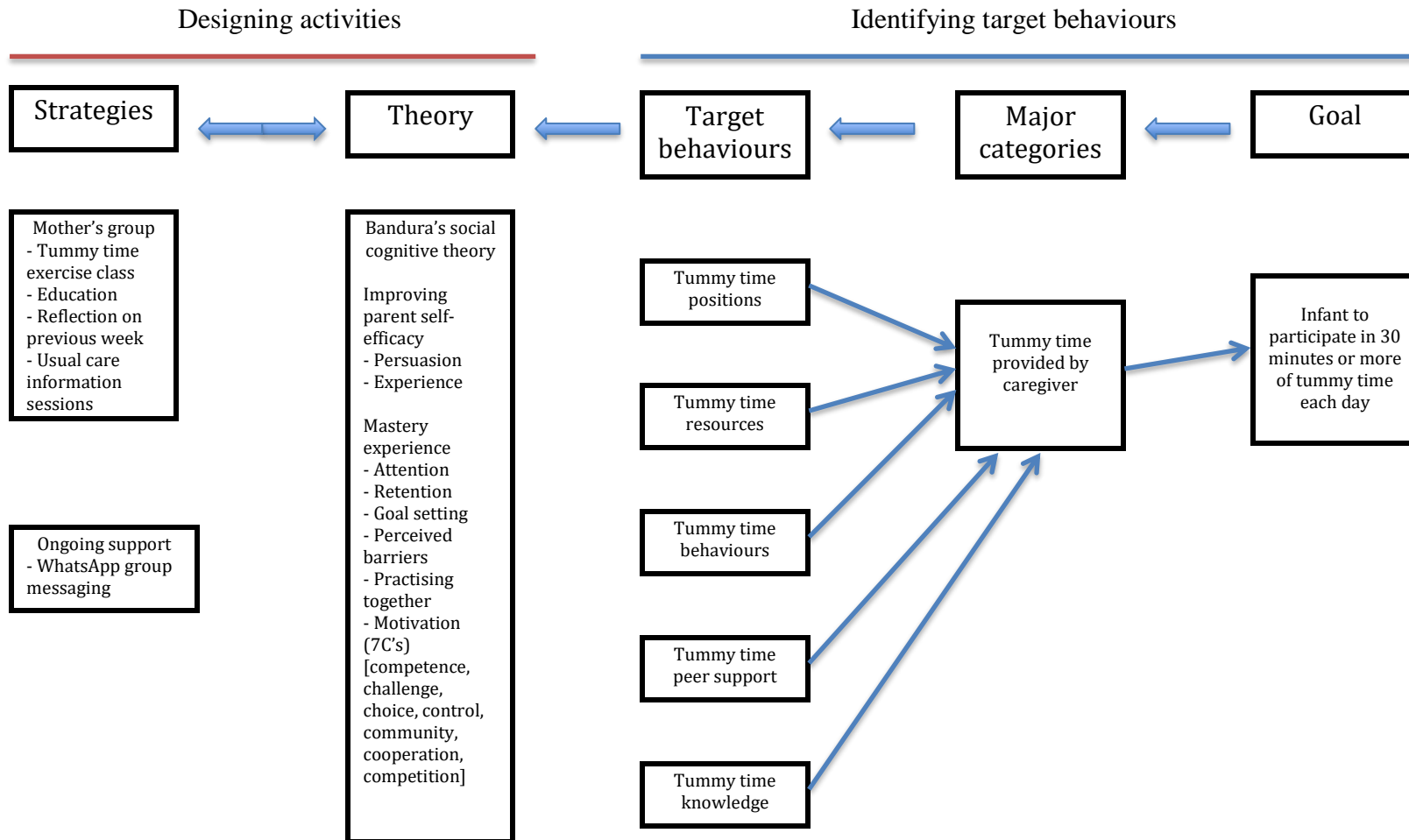

Supplement: Supplementary file 1 — Additional file 1: Bandura’s social cognitive theory – Intervention mapping [file 40814_2020_695_MOESM1_ESM.pdf]
