# Supplementary material for: Weekly group tummy time classes are feasible and acceptable to mothers with infants: a pilot cluster randomized controlled trial
Source: Pilot Feasibility Stud. 2020 Oct 14;6:155. doi: 10.1186/s40814-020-00695-x (PMC7556919; doi:10.1186/s40814-020-00695-x)
Supplement: Supplementary file 2 — Additional file 2: Usefulness and relevance questionnaires [file 40814_2020_695_MOESM2_ESM.pdf]

EARLY START | UNIVERSITY OF WOLLONGONG AUSTRALIA | NSW GOVERNMENT Health Illawarra Shoalhaven Local Health District

<u>Perceived usefulness of components of the physical activity intervention</u>

How useful was:

1. The information overall:

☐ ☐ ☐ ☐ ☐ ☐
Extremely Very Moderately Slightly Not at all

Other comments:

2. The goal planning:

☐ ☐ ☐ ☐ ☐ ☐
Extremely Very Moderately Slightly Not at all

Other comments:

3. The handouts given at mother's group:

☐ ☐ ☐ ☐ ☐ ☐
Extremely Very Moderately Slightly Not at all

Other comments:

4. The WhatsApp group messages sent by researcher:

☐ ☐ ☐ ☐ ☐ ☐
Extremely Very Moderately Slightly Not at all

Other comments:

5. The WhatsApp group messages sent by other group members:

☐ ☐ ☐ ☐ ☐ ☐
Extremely Very Moderately Slightly Not at all

Other comments:

6. The WhatsApp to provide social interaction and peer support between group members:

☐ ☐ ☐ ☐ ☐ ☐
Extremely Very Moderately Slightly Not at all

Other comments:

7. The face-to-face mother's group to provide social interaction and peer support between group members:

☐ ☐ ☐ ☐ ☐ ☐
Extremely Very Moderately Slightly Not at all

Other comments:

8. The tummy time practice as a group:

☐ ☐ ☐ ☐ ☐ ☐
Extremely Very Moderately Slightly Not at all

Other comments:

EARLY START | UNIVERSITY OF WOLLONGONG AUSTRALIA | NSW GOVERNMENT Health Illawarra Shoalhaven Local Health District

<u>Perceived relevance of components of the physical activity intervention</u>

How relevant was:

1. The information overall:

☐ Extremely ☐ Very ☐ Moderately ☐ Slightly ☐ Not at all ☐

Other comments:

2. The goal planning:

☐ Extremely ☐ Very ☐ Moderately ☐ Slightly ☐ Not at all ☐

Other comments:

3. The handouts given at mother's group:

☐ Extremely ☐ Very ☐ Moderately ☐ Slightly ☐ Not at all ☐

Other comments:

4. The WhatsApp group messages sent by researcher:

☐ Extremely ☐ Very ☐ Moderately ☐ Slightly ☐ Not at all ☐

Other comments:

5. The WhatsApp group messages sent by other group members:

☐ Extremely ☐ Very ☐ Moderately ☐ Slightly ☐ Not at all ☐

Other comments:

6. The WhatsApp to provide social interaction and peer support between group members:

☐ Extremely ☐ Very ☐ Moderately ☐ Slightly ☐ Not at all ☐

Other comments:

7. The face-to-face mother's group to provide social interaction and peer support between group members:

☐ Extremely ☐ Very ☐ Moderately ☐ Slightly ☐ Not at all ☐

Other comments:

8. The tummy time practice as a group:

☐ Extremely ☐ Very ☐ Moderately ☐ Slightly ☐ Not at all ☐

Other comments:

Perceived usefulness of components of the mother's group

How useful was:

1. The information overall:

☐	☐	☐	☐	☐
Extremely	Very	Moderately	Slightly	Not at all

Other comments:

2. The goal planning:

☐	☐	☐	☐	☐
Extremely	Very	Moderately	Slightly	Not at all

Other comments:

3. The handouts given at mother's group:

☐	☐	☐	☐	☐
Extremely	Very	Moderately	Slightly	Not at all

Other comments:

4. The face-to-face mother's group to provide social interaction and peer support between group members:

☐	☐	☐	☐	☐
Extremely	Very	Moderately	Slightly	Not at all

Other comments:

5. Was there a social media group set up between mothers? (Example, WhatsApp, Facebook etc)

☐	☐
Yes	No

Other comments:

6. If there was a social media group set up between mothers, how did you perceive the usefulness of this group?

☐	☐	☐	☐	☐
Extremely	Very	Moderately	Slightly	Not at all

Other comments:

Perceived relevance of the mother's group

How relevant was:

1. The information overall:

☐	☐	☐	☐	☐
Extremely	Very	Moderately	Slightly	Not at all

Other comments:

2. The goal planning:

☐	☐	☐	☐	☐
Extremely	Very	Moderately	Slightly	Not at all

Other comments:

3. The handouts given at mother's group:

☐	☐	☐	☐	☐
Extremely	Very	Moderately	Slightly	Not at all

Other comments:

4. The face-to-face mother's group to provide social interaction and peer support between group members:

☐	☐	☐	☐	☐
Extremely	Very	Moderately	Slightly	Not at all

Other comments:

5. Was there a social media group set up between mothers? (Example, WhatsApp, Facebook etc)

☐	☐
Yes	No

Other comments:

6. If there was a social media group set up between mothers, how did you perceive the relevance of this group?

☐	☐	☐	☐	☐
Extremely	Very	Moderately	Slightly	Not at all

Other comments:
